# Students’ persistence intention in MOOCs in the psychomotor domain: An extended 3P model of the teaching and learning perspective

**DOI:** 10.3389/fpsyg.2023.1094138

**Published:** 2023-02-27

**Authors:** Hsi-Hsun Yang, Jia-Yu Lin

**Affiliations:** Department of Digital Media Design, National Yunlin University of Science and Technology, Douliu, Taiwan

**Keywords:** psychomotor domain, MOOCs, 3P model of teaching and learning, structural equation modeling, persistence intention

## Abstract

This study proposed and tested a model adapted from Biggs’ 3P model that quantifies the behaviors of students who completed MOOCs (Massive Open Online Courses) in order to design intervention measures for low retention rates. Psychomotor domain data from 300 MOOC learners was analyzed in a covariance-based structural equation model (CB-SEM) to analyze the direct and indirect effects of various factors. Results show the basic psychological needs theory (BPNT) in the presage stage significantly positively correlated with engagement in the process stage. Meanwhile, the process stage exhibited a significantly positive correlation with the product stage, representing persistence intention (PI). Furthermore, a full mediation effect was observed among the presage, process, and product stages. The mediating effect demonstrates that higher student engagement leads to more positive exertion on BNPT and PI to complete the course. Moreover, results show bolstering students’ behavioral, emotional, and cognitive engagement strengthens their PIs.

## 1. Introduction

The average dropout rate of students who take MOOCs has been reported as high as 90% (Vitiello et al., 2018; Xing and Du, 2019). Such a low completion rate has raised concerns among course providers, educators, and researchers (Cobos and Ruiz-Garcia, 2020; Dai et al., 2020). While most researchers have focused on learner behaviors, cognitive and intentions (Wong et al., 2019; Lambert, 2020; Li and Zhao, 2021; Tao et al., 2022), regardless of courses type, the current study asserts that MOOCs including psychomotor topics should be discussed independently. The difficulty in implementing courses in the psychomotor domain lies in teachers’ inability to directly supervise learners in person. Therefore, this study addressed this gap by focusing on such courses–account for ~10% of all MOOCs. The online learning modes of such courses have evolved along with technology advances. The psychomotor domain has been explored by a few scholars (Yang and Su, 2017; Tien et al., 2020); however, such studies are not supported by extensive research evidence or widely reported. Although this type of MOOC is in the minority of class designs (Shah, 2021). By better understanding the mechanisms of how students in a psychomotor domain class decide to learn, engage with the course, and persist to completion, improvements can be made to course design and by implication student completion and uptake.

Badali et al. (2022) reviewed attempts to add new constructs based on classic theoretical models, such as the technology acceptance model, self-determination theory, expectation confirmation theory, theory of planned behavior, and unified theory of acceptance and use of technology. Such previous work attempted to identify influencing factors of intentions to keep learning among students, pointing out attention should be paid to phased and persistent behaviors among students when learning. The learning process (e.g., timelines) should be incorporated to better identify elements affecting learning behaviors across cause, process, and result. Taking this into consideration, Biggs’ 3P (presage, process, and product stages) model has been modified into a classic model with fixed elements (de la Fuente et al., 2015; Chan and Yeung, 2020; Deng et al., 2020). Kanashiro et al. (2020) surveyed 46 publications related to the 3P model from 2002 to 2017. The authors highlighted that despite criticism for being outdated and oversimplified, the 3P model appears to be the most prominent in higher education by showing that the teaching context and students’ background (presage factors) affect students’ approach to learning (process stage), which can range from surface learning to deep learning (product stage). The classic model gathers all phenomena and covers all influence factors based on timelines. Such factors are not constant and can adapt according to the actual research environment that contributes to examining the causes of a certain teaching or learning phenomenon (Albelbisi et al., 2018; López-García et al., 2019). However, the 3P theoretical model has not been universally adopted for solving the problem of low MOOC completion rates within the psychomotor domain. Therefore, this research gap needs to be addressed, which we discuss next.

In determining a student’s motivation to enroll in a MOOC, the learner’s internal motivation of pleasure and expectation of personal accomplishment should be first quantified (Li et al., 2013; Rump et al., 2017), which also involves satisfying their basic psychological needs (Sun et al., 2019; Ryan and Deci, 2020). The Basic Psychological Needs Theory (BPNT) posits human behaviors are under the influence of incentives of internal motivation. BPNT highlights three basic psychological needs: perceived autonomy, perceived competence, and perceived relatedness, which are the common needs everyone is born with (Deci and Ryan, 1985; Dincer et al., 2019) and have been widely discussed in various online learning contexts by many researchers (Jeno et al., 2019; Hsu, 2021). Satisfying students’ basic psychological needs is considered a prerequisite of classroom engagement (Reeve, 2012), while engagement is regarded as a prerequisite to learning (Guo et al., 2014), which in turn affects the completion rate of students (Appleton et al., 2008). It is worth mentioning that behavioral, emotional, and cognitive engagement are dynamically interrelated, rather than isolated processes (Fredricks et al., 2004). The rare combination of the 3P theory, BPNT, and engagement is also worth exploring; hence, the second key point of this study. Based on the above premise, this study employed persistence intention (PI), as specified by Jung and Lee (2018), as an indicator of students’ completion of courses. It is valuable to probe the relationships among basic psychological needs, classroom engagement, and student PI.

In this regard, the current study integrated the learning contexts of MOOCs into Biggs’ theoretical model. As such, the presage stage stands for BPNT, the process stage stands for engagement, and the product stage stands for PI. The current study has several objectives. The first objective is to quantify the factors—as well as their mechanisms—that affect students’ behavior to persist in finishing MOOCs during the entire learning process. The second is to qualify how members recognize the presage stage of psychomotor MOOCs while examining how such recognition may affect persistence intention. Furthermore, this research explored the process stage of MOOCs and examined the effects on the links between the presage stage and persistence intention.

## 2. Hypothesis development

In Bloom’s taxonomy of educational objectives (Bloom et al., 1956; Andreatta and Dougherty, 2019), educational activities in the psychomotor domain focus more on fostering students’ psychomotor abilities (Harrow, 1972). Psychomotor ability is the ability to control motor actions proceeding a psychological process (including brain activity). Studies have shown that courses in the psychomotor domain can help students learn to make clothes, perform surgical operations, machine materials, cook, and master other psychomotor activities (Andreatta and Dougherty, 2019; Tien et al., 2020).

Studies using Biggs’s (1993) model of students’ learning approaches suggested three stages—the presage-process-product (3P) mode in non-traditional learning environments. They propose interaction among factors forms a dynamic learning model that influences learning outcomes (Lee and Chan, 2018). These three stages represent pre-, learning in, and post-learning (Biggs and Tang, 2003). The presage stage emphasizes that the teachers are central to the learning process, and building the sustainability and legitimacy of teachers requires a combination of personal beliefs and external verification (Maloni et al., 2012). In addition, on the student side, it highlights the importance of the student’s experience and expectations for learning approaches. Furthermore, it requires constant educational institutional support (Barth, 2013). The process stage describes how teaching and students’ experience are integrated, and teaching effectiveness appears to be a critical factor in this stage (Kanashiro et al., 2020), such as classroom engagement (Pilli and Admiraal, 2017). The final stage – product, generally refers to any outcome related to teaching and learning. The assessment of learning outcomes may be complex but can be considered in quantitative and qualitative methods, such as academic achievement, dropout or completion of schooling (Pilli and Admiraal, 2017; Deng et al., 2020).

Taking a MOOC learning experience as the main factor in the presage stage (BNPT), this study found that the basic psychological needs for perceived autonomy can be satisfied by allowing students to browse course units or develop their own learning plans at will. This way, the students are allowed to be in control when studying in a MOOC context. As a result, a learner’s basic psychological need for perceived competence can be met, and such a student can share their knowledge with or seek help from lecturers and other students, addressing their basic psychological need for perceived relatedness (Lan and Hew, 2018). Once those three psychological needs are met, students will have a strong basis for motives that make them more active in learning behavior (Deci and Ryan, 1985; Ryan and Deci, 2017; Dincer et al., 2019). Taking MOOC learning engagement as the main factor in the process stage, this study consists of three interrelated components: behavioral, emotional, and cognitive engagement (Reeve and Tseng, 2011). The current study found that through the three categories of engagement, all possible types of engagement could be explained from a more comprehensive perspective (Reeve, 2012). Further, according to Núñez and León (2019), if students’ basic psychological needs are satisfied, they will be more active in taking part in learning activities. As a result, those three needs are considered sufficient conditions for learner engagement behaviors. Moreover, Benlahcene et al. (2020) indicated students’ psychological needs for perceived competence and perceived relatedness could be used to predict engagement in the process stage. Therefore, the current research proposed the following hypothesis:

*H1*: The presage stage (BPNT) positively affects the process stage (engagement).

This study set MOOC learning performance as the critical factor in the product stage (persistence intention). According to Khalil and Ebner (2014), when a MOOC stimulates positive motivations to learn, the proportion of learners who keep learning increases. Among those motivations, the internal motivation of learning is more effective. Hence, cultivating internal motivation was considered an excellent method to stimulate students to complete their courses (Xiong et al., 2015). The quality of internal motivation is mainly influenced by the positive effects of a learner’s basic psychological needs (Sørebø et al., 2009; Han et al., 2017). Assuming these basic needs are satisfied, students’ internal motivations positively correlate with their intentions to complete a MOOC (Han et al., 2017). Therefore, the interaction of these factors determines learning outcomes–referred to as the product. Thus, this research proposed the following hypothesis:

*H2:* The presage stage (BPNT) positively affects the product stage (persistence intention).

Classroom engagement has been proven to be an effective predictor of persistent learning (Jung and Lee, 2018; Dincer et al., 2019). Furthermore, Oh and Lee (2016) and Yu et al. (2020a,b) pointed out that the dominant contributory factor is a prerequisite to persistent learning. Similarly, Dincer et al. (2019) found cognitive engagement is negatively correlated with dropout rate, implying higher cognitive engagement leads to lower dropout rates. Therefore, this study proposed the following hypothesis:

*H3:* The process stage (engagement) positively affects the product stage (persistence intention).

Previous research has generally confirmed that perceived autonomy, competence, and relatedness are at the core of learning motivation through persistence intentions (Sun et al., 2019). When BPNT puts members in a good learning motivation environment, it fosters both their engagement and persistence intention. In other words, there is a relationship among the three basic psychological needs, engagement, and persistence intention, in which engagement serves as the link between the three basic psychological needs and persistence intention. The individual’s basic psychological preferences affect both the member’s engagement and persistence intention (Jung and Lee, 2018). Thus, a learner’s engagement affects their persistence intention (Yu et al., 2020a,b). Therefore, we hypothesized that engagement would play a mediating effect between the three basic psychological needs and persistence intention. In addition, based on the Motivation Theories on Learning, engagement can contribute to a learner’s persistence intention by fostering a long-lasting learning motivation (Lerdpornkulrat et al., 2018). Therefore, the following hypothesis is proposed:

*H4:* The process stage (engagement) mediates the association between the presage stage (BPNT) and the product stage (persistence intention).

## 3. Materials and methods

### 3.1. Research framework

**Figure 1:**
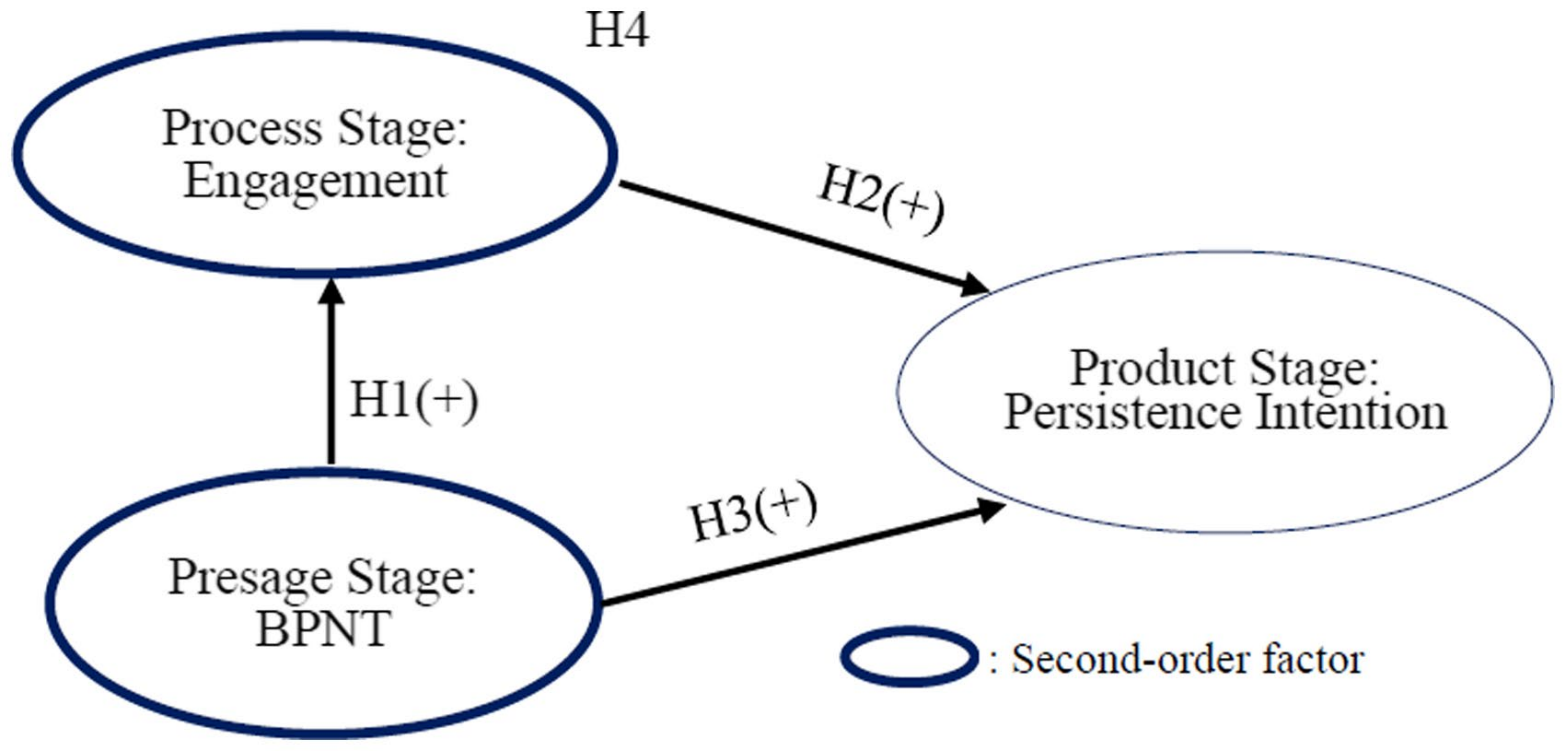
Research framework.

The current research proposed that the presage stage (including perceived autonomy, perceived competence, and perceived relatedness) positively impacts the process stage (including behavioral, emotional, and cognitive) and the product stage (including persistence intention). Meanwhile, the process stage mediates the association between the presage stage and the product stage (Figure 1).

### 3.2. Measures

The first part of the questionnaire includes four demographic questions about age, gender, educational background, and the times of logging into the MOOC platform every week. The second part of the questionnaire has 10 questions about perceived autonomy, competence, and relatedness to basic psychological needs. Further, based on Standage et al. (2005), and Reeve and Sickenius (1994), 15 questions on behavioral, emotional, cognitive, and agentic engagement were included (Skinner et al., 2008, 2009; Mameli and Passini, 2019). An additional three questions about persistence intention in completing MOOCs were also included based on Kalz et al. (2015) to measure students’ views on these seven potential variables when taking psychomotor domain courses on the learning platform. The questionnaire was designed using a seven-point Likert formula to measure the interviewees’ degree of consent to each question.

### 3.3. Subjects and data collection

Deleting invalid responses resulted in a total of 300 valid questionnaires. Participants all attended MOOC courses offered by the TaiwanLIFE and Ewant learning platforms. These courses included one course in food processing (with a sample size of 44), three courses in cooking (with a sample size of 20, 33, and 29, respectively), three courses in basic and intermediate 2D animation (with a sample size of 69, 41, and 66 respectively), and one course in Chinese calligraphy (with a sample size of 4). Common across these courses is their psychomotor component that requires learners to practice while learning by observing and imitating the behavior of teachers and sharing their finished work made with their own hands. The instructional duration of each course ran from 9–13 weeks. In the middle stage of the courses (weeks 4–6), researchers released online questionnaires through the course’s bulletin board and gave participants 2 weeks to complete. Leaners completed the questionnaires voluntarily with no impact on their grades.

The SPSS version 25 software was employed to perform descriptive statistics on the questionnaire demographic section. Results showed 72 (24.0%) participants were male and 228 (76.0%) female. The largest number of participants, 98, were under the age of 20 (32.7%), followed by 77 (25.7%) aged 21–30, and 53 (17.7%) aged 41–50. In terms of educational background, 141 (46.7%) participants graduated or were enrolled in a high school or vocational high school, followed by 82 (27.3%) who graduated or were enrolled in a university or college. Weekly logging into the MOOC showed 146 (48.7%) learners logged in 1–2 times, 79 (26.3%) learners logged in 3–4 times, and 48 (16.0%) logged in 5–6 times.

### 3.4. Data analysis

The two-step approach of structural equation modeling (SEM) was suggested by Anderson and Gerbing (1988) to estimate the measurement model and structural analysis. Descriptive analysis was used to describe the profile of the participants, and path analysis was then applied to test the relationships among the independent variables of the presage, process, and product stages, including the mediating effects of the process stage. Structural equation modeling using AMOS version 24 was employed to establish the nature of the relationship between the independent and dependent variables. With the use of the maximum likelihood estimation (MLE), a confirmatory factor analysis of the fit was conducted. Next, a relative path analysis was combined to define the model. All statistical analyses were conducted at a significance level of *p* ≤ 0.05.

## 4. Empirical analysis and results

### 4.1. Confirmatory factor analysis

As shown in Table 1, the composite reliability values of the constructs ranged from 0.864 to 0.948, exceeding the 0.7 value recommended by Nunnally and Bernstein (1994) and indicating that all constructs are internally consistent. All average variances extracted (AVE) ranged from 0.674 to 0.845, exceeding the suggested level of 0.5 (Hair et al., 2010) and demonstrating adequate convergent validity.

**Table 1:** Reliability and validity analysis results.

Construct	Construct reliability	Convergent validity	Measurement variable						
	CR	AVE	PA	PC	PR	BE	CE	EE	PI
PA	0.892	0.674	**0.821**						
PC	0.933	0.822	0.751	**0.908**					
PR	0.921	0.796	0.574	0.644	**0.892**				
BE	0.864	0.682	0.722	0.672	0.606	**0.827**			
CE	0.942	0.845	0.582	0.471	0.392	0.683	**0.919**		
EE	0.948	0.819	0.639	0.555	0.474	0.787	0.843	**0.906**	
PI	0.931	0.820	0.636	0.485	0.441	0.767	0.618	0.630	**0.905**

PA, perceived autonomy; PC, perceived competence; PR, perceived relatedness; BE, behavioral engagement; CE, cognitive engagement; EE, emotional engagement; PI, persistence intention.

For each construct to be discriminately valid, the square root of the average variance extracted (AVE) of a given construct must be larger than its correlations with any other constructs (Hair et al., 2010). As shown in Table 1, all bold numbers in the diagonal direction, representing the square roots of AVE of each construct, are larger than the correlations with other constructs, supporting discriminant validity.

### 4.2. Model fit

When there are more than 200 samples in SEM, the chi-square value (*χ*^2^ = (*n* − 1) FMIN) may be too large. FMIN refers to the minimum value of the difference between the sample matrix and the expectation matrix. When a chi-square value is too large due to an excessive number of samples, the value of *p* is likely to be considered unacceptable (Hsu and Yen, 2013). Therefore, based on the suggestions of Bollen and Stine (1992), this study adopted the Bootstrap method to revise the chi-square value from 670.844 to 357.336 and re-estimate it. As shown in Table 2, the obtained major indices of model fit are *χ*^2^/DF = 1.412 < 3; RMSEA = 0.045 < 0.08; GFI = 0.950 > 0.9; AGFI = 0.934 > 0.9; NFI = 0.950 > 0.9; TLI (NNFI) = 0.978 > 0.9; CFI = 0.980 > 0.9. All indices showed a good level of model fit. A more detailed illustration is presented in the Discussion section.

**Table 2:** Model fit indicators.

Model fit	Criteria	Model fit research model
*χ* ^2^	The small the better	357.336
DF	The large the better	253
Normed Chi-sqr (*χ*^2^/DF)	1 < *χ*^2^/DF < 3	1.412
RMSEA	<0.08	0.045
GFI	>0.9	0.950
AGFI	>0.9	0.934
NFI	>0.9	0.950
TLI(NNFI)	>0.9	0.978
CFI	>0.9	0.980

### 4.3. Analysis

**Figure 2:**
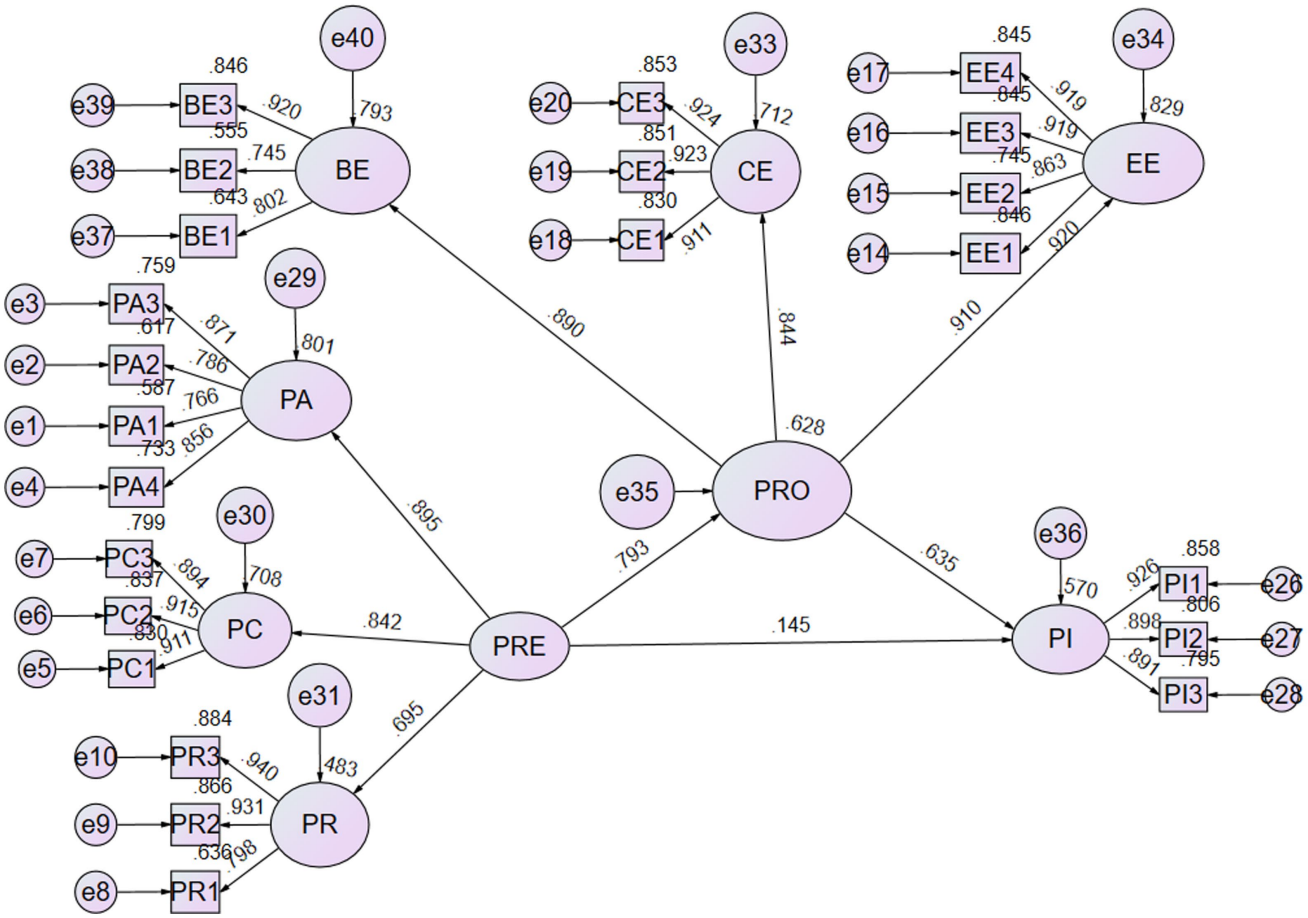
Path analysis stage of presage, process, and product.

Path analysis results show the presage stage (*β* = 0.793, *t* = 9.193) significantly affected perceived value. The process (*β* = 0.635, *t* = 6.043) and presage stages (*β* = 0.145, *t* = 1.356) significantly affected persistence intention. These findings support H1 and H3. As shown in Table 3 and Figure 2, the presage stage (*β* = 0.145, *t* = 1.356) had no significant impact on persistence intention; hence, H3 is not supported. Lastly, the mediating effect (*β* = 0.56, *t* = 9.193) among the presage stage, the process stage, and PI was significantly positive. Therefore, H4 is supported.

**Table 3:** Path relationship test table.

Path	Standardized estimated	*t*	Value of *p*	Bias-corrected CI 95%		*R* ^2^	Hypothesis supported
				Lower bound	Upper bound		
H1: PRE→PRO	0.793	9.193	***	0.349	0.923	0.628	Yes
H2: PRO→PI	0.635	6.043	***	0.251	1.016	0.570	Yes
H3: PRE→PI	0.145	1.356	0.175	0.145	−0.218	–	No
H4: PRE→PRO→PI	0.533	2.429	**	0.179	0.993	–	Yes

**p < 0.01; ***p < 0.001.

To sum up, owing to the significant indirect effects (H4) and the insignificant direct effects (H3), this type of mediation can be classified as full mediation as maintained by previous scholars (Baron and Kenny, 1986). In other words, the process stage strongly affects the presage stage and persistence intention. Besides, this study also found the values of R2 of the process stage and persistence intention were 0.628 and 0.570, respectively. Meanwhile, according to the recommended standard from Chin (1998), the value of R2 between 0.33 and 0.67 represents a moderate explanatory power. Therefore, the model in this study exhibits moderate explanatory power.

Perceived autonomy is most strongly associated with learners’ basic psychological needs (coefficient of 0.895), followed by perceived competence (0.839) and perceived relatedness (0.697). On the other hand, emotional engagement contributes the most to the learners’ engagement (coefficient of 0.942), followed by cognitive engagement (0.884) and agentic engagement (0.591), as shown in Figure 2.

### 4.4. Mediation analysis

To examine whether the process stage mediates the effect of the presage stage on MOOCs’ persistence intention, this study conducted a mediation analysis using the bootstrapping method proposed by Preacher and Hayes (2008). Bootstrapping has been widely employed in mediation analysis to determine whether the relationship between an independent and a dependent variable is due to the mediating variables—either fully or partially. The effect of each potential variable is presented in Table 2. First, PRE showed both direct (*β* = 0.318, *p* < 0.05) and indirect (*β* = 0.330, *p* < 0.05) effects on persistence intention. Thus, the total effect of the presage stage on persistence intention was *β* = 0.648 (*p* < 0.05), indicating the significance of the indirect effect of the process stage on the relationship between the presage stage and persistence intention. Second, the direct effect size of the presage stage on persistence intention was 0.452. The presage stage had a direct positive effect on the process stage (*β* = 0.730, *p* < 0.05). Third, the process stage had a direct positive effect on persistence intention as well (*β* = 0.318, *p* < 0.05).

## 5. Discussion and conclusions

### 5.1. Index of model fit

The chi-square value is generally used as an index of the absolute fit between the model and data. One example of a statistic that minimizes the impact of sample size on a better model fit is *χ*^2^/DF (Wheaton et al., 1977). While there is no consensus on an acceptable ratio for this statistic, the suggested range is from 5.0 (Wheaton et al., 1977) to 2.0 (Tabachnick and Fidell, 2007); 3.0 was selected for this study (Kline, 2005). Meanwhile, the chi-square value of this model was 1.579, suggesting a good fit.

Other indices of the absolute fit index include the goodness-of-fit (GFI), adjusted goodness-of-fit (AGFI), and root mean square error of approximation (RMSEA). The GFI and AGFI describe the proportion of variance accounted for by the estimated population covariance, analogous to R2. The closer the GFI and AGFI value is to 1, the larger the value indicates a better fit; otherwise. Generally, researchers suggest that a GFI and AGFI value >0.9 indicates a good fit (Hu and Bentler, 1999). The GFI and AGFI values of the current model are 0.949 and 0.934, respectively, showing the data match the model well. According to its definition, the RMSEA measures the discrepancy due to the approximation per degree of freedom (Steiger, 1989). MacCallum et al. (1996) used 0.01, 0.05, and 0.08 to indicate excellent, good, and mediocre fit, respectively, while the RMSEA of the current model is 0.044—somewhat below the good fit level of 0.05.

Another index used to assess model fit is the incremental fit index, which compares a chi-square for the model with a null model (also called a baseline model or independence model). There are several incremental fit indices, including the comparative fit index (CFI), normed fit index (NFI), and Tucker-Lewis index [TLI, also called the non-normed fit index (NNFI)]. All of these indices exhibited values ranging between ~0 and 1.0. However, the CFI, NFI, and TLI values were between 0.90 and 0.95, which are now considered marginal—above 0.95 is good and below 0.90 is considered a poor fit (Hu and Bentler, 1999). The CFI, NFI, and TLI of the current model were 0.981, 0.949, and 0.978, respectively. CFI and TLI were more significant than the traditional standard. Though NFI did not reach the high standard level of 0.95, it met the recommended value of Bentler and Bonett (1980), which should be >0.9. Therefore, NFI also met the acceptable fit threshold. In sum, these results show the model is a fair or reasonable representation of the data.

### 5.2. Academic implications

This study quantified how psychological needs of MOOC learners, in a psychomotor domain, significantly affected engagement and persistence intention. In other words, addressing those psychological needs in the first encounter between learners and the online course is key to learner success. This finding is especially applicable to courses in the psychomotor domain where students are object-oriented, such as courses focused on skills with a physiological component. When students’ autonomy, competence, and relatedness are encouraged, their internal motivations are stimulated. As a result, they participate in learning activities across behaviors, emotions, and cognition (Lan and Hew, 2018; Núñez and León, 2019).

Noteworthily, the influence of Perceived Autonomy (PA), as shown in Figure 2, is greater than Perceived Competence (PC) and Perceived Relatedness (PR). Thus, if a course intends to encourage students to present PA, such as sharing experiences, asking questions, expressing opinions, and creating study-related topics, instructors should supply positive comments to learners, which encourages the positive cycle of autonomous learning. Figure 2 also shows the influence of Emotional Engagement (EE) is greater than Cognitive Engagement (CE) and Behavioral Engagement (BE), demonstrating that BNPT encourages emotional engagement. Therefore, instructors and online instructional videos should provide a sense of emotional communication, such as occasional synchronous online classes to promote emotional interactions with and among learners (Marulanda, 2022). Our results show such activity would be beneficial in improving learner engagement. Furthermore, engagement has a positive effect on persistence intention. Such results are consistent with the previous findings of Oh and Lee (2016), Dincer et al. (2019) and Yus’ et al. (2020a,b). For example, students’ experiencing negative emotions (i.e., due to repeated failures or learning burnout) should be encouraged by instructors connecting the knowledge they learned with the experience they gained.

According to the mediating effect, BNPT directly influences persistence intention through engagement. Students are thus willing to persist in completing courses when the learning environment satisfies their BNPT and their engagement is encouraged. In other words, the three basic psychological needs of learners should be met first. Doing so encourages participation in learning activities, such as viewing instructional videos, participating in discussions, submitting homework, and completing tests. Such learning behaviors also include their attention and efforts. When students respond to their courses with positive emotions, including enjoyment, affection, fun, flow, and immersion, their emotional engagement improves. Many studies support the role of engagement as a mediator on the relationship between basic psychological needs and continuous learning behaviors (Skinner et al., 2008, 2009; Dincer et al., 2019).

### 5.3. Practical implications

Engagement centers on experience and interaction, exhibited through interactions between learner and learning environment (Lan and Hew, 2018). Teachers can support the three basic psychological needs and assist students in building their confidence. In terms of perceived autonomy, teachers should try to avoid a domineering teaching style in which students are required to complete learning activities within a short period (1–2 weeks; Alamri et al., 2020; Chiu, 2021). Indeed, it is difficult to manage and control homework submissions in the MOOC design considering the high level of learner autonomy. One of the participating MOOC instructors (teaching the animation courses) participating in this study required homework submissions on schedule with missed deadlines resulting in score deductions (Ihantola et al., 2020; Denny et al., 2021). Yet, when a more flexible practice was adopted, where students could submit their homework before the course completion, the submission rate and the quality of homework was higher.

We have observed three different interactions. First, student-content interaction is mainly based on the performance of students. This interaction involves “behavioral engagement” and “cognitive engagement.” From the “behavioral engagement “in the questionnaire, we know that the highest score is “When I watch the intructional martial video, I will concentrate on listening to the content.” A score of 6.21 out of 7, which can reflect the learners’ attitude toward video views, course access, and reading text satisfaction, especially video watching is the most valued and used resource for MOOC learners (Gregori et al., 2018). In addition, from the “cognitive engagement” in the questionnaire, the highest score is “When I study this course, I will try to connect what I have learned with my experience.” A score of 6.21 out of 7. In general, multimedia can stimulate students’ interest and promote course retention and learning.

Second, student–student interaction is mainly about students seeking help, peers’ feedback, and students share for other resources. These behaviors can be seen in one of the courses we investigated, the 2D animation series courses, where students will share notes and experiences. And some students volunteer to assist teachers in answering questions. There is another cooking course, where students are primarily working people. They have the same background and social experience, so the issues discussed can resonate.

Third, student-lecturer interaction is mainly for teachers to provide emotional support and encouragement to students. The specific method is like teachers posting information in the discussion area or answering students’ questions. This interaction involves “Emotional Engagement.” From the “emotional engagement” in the questionnaire, the highest score is “I like to learn new knowledge in this course.” With a score of 6.25 out of 7. Teachers can release more autonomy to students, helping students succeed in learning, building self-confidence, and creating a positive classroom atmosphere.

The question, “I am close to the teaching assistant of this course,” in the questionnaire received the lowest score of 4.92 (of a maximum 7). Given the basic need for perceived relatedness, this finding implies that two to three live broadcasts can be arranged during the courses to compensate for such a lack of close contact. Synchronous online sessions supply instructors an opportunity to exhibit interest in students’ learning and recognize their performance (Phan et al., 2016; Vollet et al., 2017). More attention should be paid to teacher-student interaction in the MOOC context when learning involves the psychomotor domain (Hew, 2014). Finally, in terms of the basic need for perceived competence supported by teachers, each learning task assigned by teachers should be linked to specific training objectives and purposes. Tasks should also adopt peer assessment accompanied by criteria to achieve learning results (Huisman et al., 2018; Gamage et al., 2021).

When designing courses in the psychomotor domain, teachers can consult with the taxonomies of educational objectives proposed by Dave (1970). Taxonomies provide five levels of practice modes for one type of exercise, which allows students to develop a skill at the levels of imitation, manipulation, precision, articulation, and naturalization (Begam and Tholappan, 2018). For instance, in the cooking courses included in this study, the cooking process was video recorded from multiple angles with the recipe displayed for demonstration. Simultaneously, a group of students were arranged to imitate the process and raise questions, helping learners feel telepresence. Furthermore, students were encouraged to comment on the learning experience, take notes and report other related experiences. In this way, learners interacted with each other, increasing engagement and improving the three basic needs. In particular, a sense of belonging from interaction effectively motivates students in the psychomotor MOOC context.

### 5.4. Limitations and future directions

This study presents some limitations. First, Biggs’ 3P model addresses how teaching and students’ contextual experiences integrate and interdependency to explain students learning outcomes. In addition to behavioral, emotional, and cognitive engagement, the process stage can add agentic engagement (Benlahcene et al., 2020; Chiu, 2021) to further explore the differences in the antecedents and consequences of different engagements.

Second, the MOOCs in the psychomotor domain in this study come from the same learning platform, so there may be a need for common method variance. It is suggested that future researchers can cover the multiple MOOCs in psychomotor domain of the platform to analyze the 3P model for persistence intention more comprehensively and accurately.

Third, most of the previous engagement research has focused on classroom observations (Jung and Lee, 2018; Dincer et al., 2019; Benlahcene et al., 2020). This study is one of the few that focuses on engagement in the MOOCs context. In the future, it should be possible to use the teaching mode of SPOCs (Small Private Online Courses) to add engagement issues, so that you can observe both the intention of online courses and the behavior in the classroom.

## 6. Conclusion

The research model presented in this study was adapted from the 3P theoretical model to clarify the behavioral processes that motivate learners to persist in completing a MOOC. This study also employed the CB-SEM method to analyze and examine relationships among BNTP in the presage stage, engagement in the product stage, and persistence intention in the product stage throughout the learning process.

Furthermore, the current study demonstrated that satisfying students’ basic psychological needs improves their engagement. Results have shown that incentives meeting psychological needs for perceived relatedness improve emotional engagement and initiative to engage. In addition, the results of the examination of the mediating effects showed that students’ engagement influenced BNPT and persistence intention. Finally, this study has proven that measures that bolster students’ behavioral, emotional, and cognitive engagement can promote their persistence intentions.

In summary, the above findings are conducive for educators, teachers, and course designers, who consider providing courses in the psychomotor domain through MOOC platforms to reassess the importance of students’ BNPT and engagement in class and encouraging persistence intentions. Meanwhile, these findings also provide a significant academic reference for online teaching strategies that improve the completion rates of online courses in the psychomotor domain.

## Data availability statement

The original contributions presented in the study are included in the article/supplementary material, further inquiries can be directed to the corresponding author.

## Ethics statement

Ethical review and approval were not required for the study on human participants following the local legislation and institutional requirements. This study was carried out following the APA Code of Ethics recommendations with written informed consent from all subjects. The patient/participants provided their written informed consent to participate in this study.

## Author contributions

H-HY: conceptualization and writing—review and editing. H-HY and J-YL: formal analysis, investigation, and writing original draft. All authors contributed to the article and approved the submitted version.

## Conflict of interest

The authors declare that the research was conducted in the absence of any commercial or financial relationships that could be construed as a potential conflict of interest.

## Publisher’s note

All claims expressed in this article are solely those of the authors and do not necessarily represent those of their affiliated organizations, or those of the publisher, the editors and the reviewers. Any product that may be evaluated in this article, or claim that may be made by its manufacturer, is not guaranteed or endorsed by the publisher.
